# Integrated microfluidics-based construction of anti-BTN2A2 gel droplet cell preparations for non-invasive tumor-infiltrating lymphocyte therapy

**DOI:** 10.1016/j.mtbio.2025.101545

**Published:** 2025-02-04

**Authors:** Yishen Tian, Jingxuan Li, Na Yang, Yang Zhao, Jiancao Zuo, Hang Xiong, Yiwen Pan, Li Xiao, Min Su, Feng Han, Zhixu He, Rong Hu

**Affiliations:** a Center for Tissue Engineering and Stem Cell Research, Guizhou Medical University, Guiyang, 550025, China; b Key Laboratory for Research on Autoimmune Diseases of Higher Education Schools in Guizhou Province, Guiyang, 550025, China; c Department of Neurosurgery, The Affiliated Hospital of Guizhou Medical University, Guiyang, 550004, China; d Department of Histology and Embryology, School of Basic Medical Sciences, Guizhou Medical University, Guiyang, 550025, China; e Department of Pediatric Hematology, The Affiliated Hospital of Guizhou Medical University, China

**Keywords:** Microfluidics chip, Gel droplets, Anti-BTN2A2, TIL therapy

## Abstract

Tumor infiltrating lymphocyte therapy (TIL therapy) is one of the effective treatments for solid tumors. However, certain periods or sites of solid tumors are not amenable to surgical resection. Meanwhile, the abundant and dense extracellular matrix (ECM) and regulatory cells (e.g., regulatory T cells (Tregs), myeloid-derived suppressor cells (MDSC)) in solid tumors when infused back into the treatment will prevent T cell infiltration and proliferation, thus inhibiting the efficacy of this approach. In this study, a novel cell preparation was successfully developed by integrating microfluidic chip design with carbodiimide chemical modification. This preparation was surface modified with BTN2A2 antibodies and internally contained T cells isolated from the blood of tumor hosts, along with simulated collagen peptide CMP. Specifically, the cell preparation exerted its anti-tumor effects through multiple mechanisms: Firstly, the surface BTN2A2 antibodies effectively inhibited the proliferation of Tregs and MDSCs within the tumor microenvironment; Secondly, leveraging the T cell antigen receptors (TCRs) present in the blood T cells, which were similar to those of tumor-infiltrating lymphocytes, significantly enhanced their targeting and cytotoxic capabilities; Furthermore, the CMP component within the droplets effectively promoted the infiltration of T cells into tumor tissues. In the complex immunosuppressive microenvironment, the synergistic action of these components markedly enhanced the clearance efficacy of the immune system. Experimental results demonstrated that this cellular preparation exhibited promising therapeutic effects in both melanoma and pancreatic cancer models. This research provided a novel platform for the synergistic cooperation of various methods in tumor immunotherapy, holding broad application prospects.

## Graphical abstract

**Figure:**
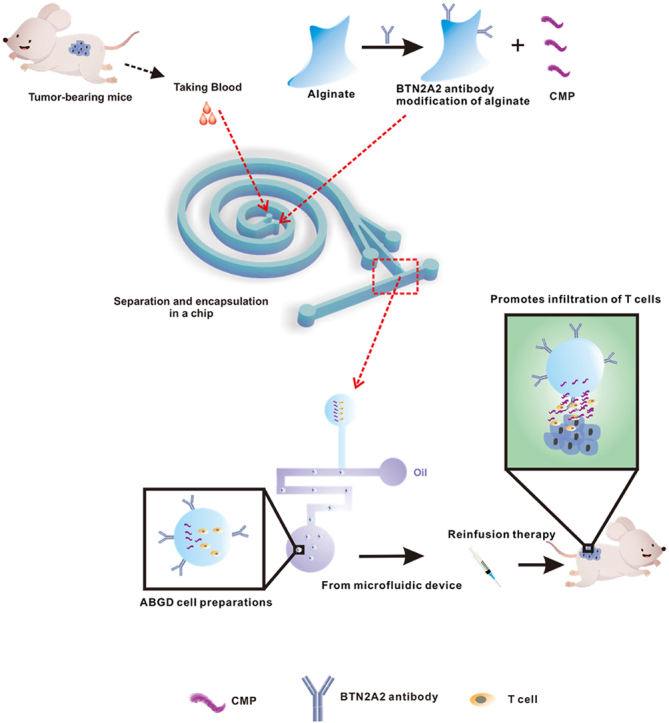

## 1 Introduction

Adoptive cell therapy (ACT) using tumor-infiltrating lymphocytes (TILs) has shown promising efficacy in clinical trials for the treatment of certain solid tumors [[1], [2], [3]]. TIL therapies offer several unique advantages in the treatment of solid tumors: (1) TILs circumvent the problem of heterogeneity in solid tumors because they consist of T cells that target multiple antigens on cancer cells. (2) Since Tumor-Infiltrating Lymphocytes (TILs) originate from the patient's own cells, their reinfusion generally does not lead to substantial adverse effects. The polyclonal nature of TILs allows them to recognize and target a wide range of patient-specific tumor neoantigens, thereby promoting the lysis of tumor cells [4]. (3) TIL therapy can be used in combination with other immunotherapies (e.g., immune checkpoint inhibitors) or chemotherapy to enhance the therapeutic effect. This combination strategy can more fully activate the patient's immune system to fight against the tumor [5,6].

The initial step in TIL therapy involves obtaining sufficient tumor tissue from the patient. For certain solid tumors, surgical resection may be the sole method to procure the necessary tumor tissue. However, not all patients are suitable candidates for or capable of undergoing surgical resection, particularly those with tumors located in complex areas or those that have extensively metastasized. Surgical resection itself carries inherent risks and potential complications, which can adversely affect the patient's overall health and subsequently interfere with the administration of TIL therapies [7,8]. The preparation process for TIL therapy encompasses the extraction of lymphocytes from tumor tissue, their subsequent expansion in vitro, as well as the execution of selection and activation steps. This procedure is both intricate and time-intensive, frequently spanning several weeks. For patients with rapidly progressing diseases, this extended timeline can pose a significant drawback. Furthermore, during the reinfusion treatment phase, solid tumors often present an immunosuppressive microenvironment, which includes immunosuppressive cells such as Tregs, MDSCs, tumor-associated macrophages (TAMs), and regulatory B cells (Bregs), among others. These elements may inhibit the activity and functionality of the TILs [9]. In summary, the realization of a non-invasive method for the acquisition of TILs, coupled with a preparation process that embodies both simplicity and efficiency while concurrently reducing costs, is crucial for enhancing the clinical feasibility of TIL therapy [10]. Moreover, if this approach can simultaneously facilitate TIL reinfusion therapy—specifically, by effectively mitigating the adverse effects of the tumor immunosuppressive microenvironment—it will further augment the clinical potential of TIL therapy.

In recent years, substantial progress has been achieved in the application of microfluidics in immunotherapy [11,12]. The use of microfluidics to enhance cellular therapies has drawn growing interest from the medical community and the public [13]. Microfluidic chips are uniquely suitable for dealing with the cellular level. Current microfluidic techniques, such as deterministic lateral displacement and inertial centrifugation, can be efficiently separated cells from blood, while microfluidic droplet technology allows for the encapsulation and cultivation of individual cells in small chambers [14,15].

In our previous work, our research group integrated microfluidic spiral inertial separation technology with gel droplet microfluidic technology to develop an innovative integrated chip capable of specific cell separation and rapid low-differentiation proliferation of single cells [16]. Utilizing this chip, we successfully isolated T cells from the peripheral blood of melanoma hosts and encapsulated them in alginate gel droplets for proliferation. Subsequently, these proliferated T cells were re-infused for therapeutic purposes, thereby establishing a non-invasive adoptive cell immunotherapy for the treatment of solid tumors. In the above-mentioned work, CD8^+^ T cells in the peripheral blood of melanoma patients could specifically recognize melanoma and had T cell antigen receptors (TCRs) similar to tumor infiltrating CD8^+^ T cells [17]. At the same time, proliferation in droplets could increase the proportion of CD8^+^ T cells (from 2 % to 12 %). However, when using T cells isolated from blood by spiral inertial separation, 15–20 % of them were Treg cells. During reinfusion therapy, both endogenous Treg cells and reinfused Treg cells jointly hindered the proliferation and infiltration of CD8^+^ T cells in the tumor, which greatly restricted the therapeutic effect. Therefore, in this study, the butyrophilin (BTN) 2A2 antibody was utilized. BTN2A2 belongs to the extended B7 family of molecules [18,19]. Studies had shown that BTN2A2 was highly expressed on the cell membranes of melanomas, gliomas, lung cancers, pancreatic cancers, and other tumors. In contrast, PD-L1 showed relatively poor expression in these tumors [20,21]. It had been discovered that the expression of BTN2A2 by tumor cells could inhibit the proliferation of activated CD8^+^ T cells and promote the proliferation of Tregs and MDSCs [22,23]. Thus, anti-BTN2A2 could promote the activation and proliferation of CD8^+^ T cells while inhibiting the proliferation of Treg cells and MDSCs.

**Scheme 1:**
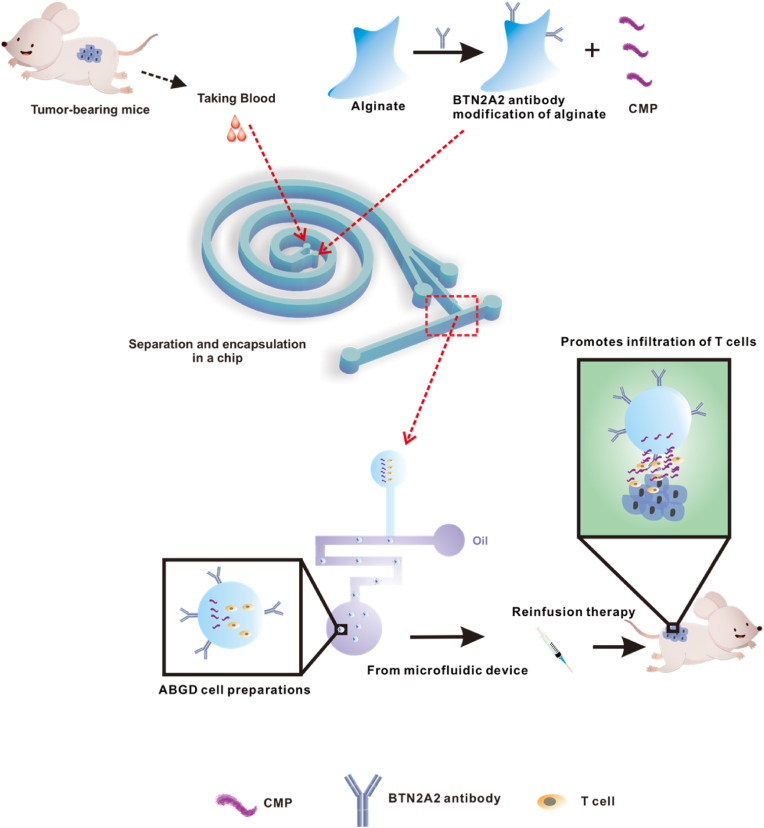
Fabrication of ABGD cell preparations based on integrated microfluidic chip and anti-BTN2A2.

Here, we employed the carbodiimide method to conjugate BTN2A2 antibodies onto alginate gels. Utilizing an integrated microfluidic chip, T cells and collagen mimetic peptides (CMP) [24] were encapsulated within anti-BTN2A2 modified gel droplets (ABGD). During re-infusion therapy, the BTN2A2 antibodies were leveraged to inhibit the proliferation of Tregs and MDSCs within the tumor. Additionally, CMP enhanced the infiltration of anti-tumor T cells into the tumor microenvironment, resulting in a favorable therapeutic outcome in the treatment of melanoma mice (Scheme 1). Building on this foundation, we applied this methodology to pancreatic cancer, which is characterized as a 'cold tumor’ due to its microenvironment being enriched with a substantial number of immunosuppressive cells, such as Tregs and MDSCs, thereby significantly limiting the infiltration and activation of CD8^+^ T cells. By employing the aforementioned strategy, T cells were directly isolated from the blood of pancreatic cancer hosts, subsequently encapsulated in droplets, and re-infused for therapy, which also exerted a certain inhibitory effect on pancreatic cancer. In summary, the combination of an integrated microfluidic chip with BTN2A2 antibodies facilitated the acquisition of an ABGD cell preparations, thereby realizing a highly efficient and cost-effective non-invasive TIL therapy.

## 2 Results and discussion

### 2.1 Designing the chip for ABGD cell preparations

**Fig. 1:**
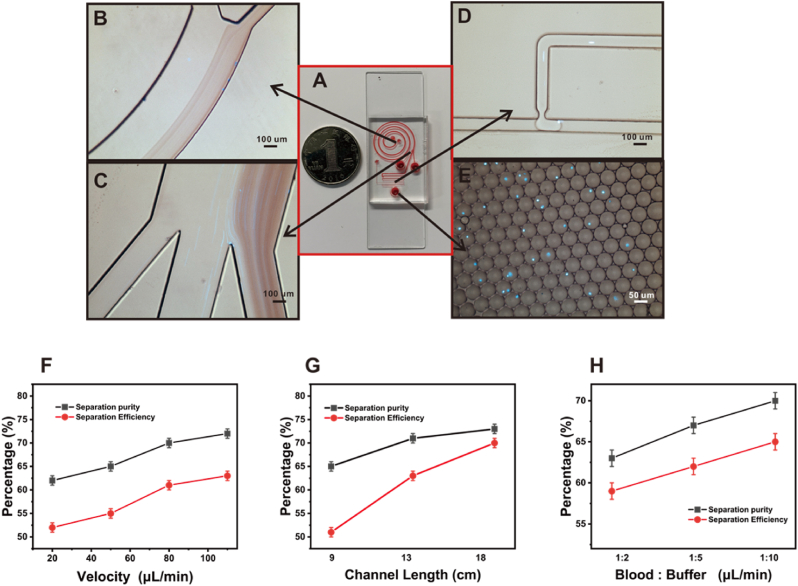
(A) Top view of the chip (channel with red ink). (B) Front section of the chip separation section. (C) Posterior section of the chip separation section with T-cell exit in the center (D) Cell encapsulation using T-junction. (E) Gel droplet encapsulation of cells. T cells were stained with Hoechst 33342. (F) Percentage of lymphocyte composition and separation efficiency at different flow velocity. (G) Percentage of lymphocyte composition and separation efficiency at different channel length. (H) Percentage of lymphocyte composition and separation efficiency at different ratios of blood and buffer.

To efficiently isolate T cells from blood and encapsulate them into gel droplets, an integrated microfluidic chip was designed (Fig. 1A). Here, the chip was designed with a separation zone (Fig. 1B and C) and a gel droplet formation zone (Fig. 1D) for efficient cell separation and encapsulation of T cells into droplets (Fig. 1E). Utilizing a spiral inertial microfluidic chip for the separation of T cells, the spiral inertial microfluidic system exhibited significant advantages in high-throughput cellular processing [25,26]. The fundamental principal hinged on the behavior of particles or cells at elevated flow velocities (with a Reynolds number, Re, ranging from 50 to 100), where they underwent lateral migration based on their size. This migration was driven by the interplay of inertial lift force (F_L_) and Dean's drag force (F_D_), resulting in the focusing of particles or cells at distinct spatial locations (Fig. S1B) [27]. As shown in Fig. 1C, T cells isolated from the bloodstream reached the droplet-forming region from the intermediate channel, and the cells were encapsulated in the droplets (Fig. 1E). According to the data from our previous experiments [16], the main influencing factors for the purity and efficiency of T cell isolation from whole blood are the flow rate, the length of the channel, and the flow rate ratio of blood to buffer. As shown in Fig. 1F, the flow rate in the chip was optimized. Increase of flow velocity results in a more significant inertial lift force (F_L_ $\propto U_{f}^{2}$) as compared to the Dean drag (F_D_ $\propto U_{f}^{1.63}$) [28]. As a result, the purity and efficiency of the separation increased as the flow rate increased. Similarly, an increased channel length leaded to a longer duration of exposure for cells to both the F_L_ and F_D_, thereby affecting a greater number of cells. This extended interaction enhanced the purity and efficiency of the separation process (Fig. 1G). In the separation region, the buffer fluid is employed to tightly constrain the blood sample, thereby preventing Dean migration and the return of small particles (e.g., platelets, etc.) to the central region of the channel [29]. Therefore, the flow rate ratio of blood to buffer also affects separation purity and separation efficiency (Fig. 1H). However, for an integrated chip, its gel droplet formation zone could limit the flow rate, length and blood/buffer flow rate ratio of the separation zone. It was not possible to select the parameters in the separation zone that would result in optimal separation purity and efficiency. Taking the experimental microchip used in this study as an example, selecting a higher flow rate in the separation section could adversely affect the subsequent formation of gel droplets. Specifically, as the aqueous phase flow rate increased, the oil phase must be subjected to a correspondingly higher flow rate to provide the necessary shear force. This could to excessive pressure within the channel, potentially preventing the formation of droplets altogether. In addition, the diameter of the droplet was affected by the flow rate of the water and oil phases. If the droplet diameter was too large, it will affect the subsequent retentate treatment experiments. Here, a flow rate of 50 (μL/min) was chosen for this experiment, with a flow rate ratio of blood to buffer of 1:10, and the length of the passage in the centrifugation section was 18 cm. In the end, 10^6^ T cells/mL were obtained from the isolated fraction. The diameter of the gel droplets was 50 μm. Since the proportion of droplets containing cells to the total droplets is in accordance with the Poisson distribution, which is determined by the concentration of the starting cells [30,31], the proportion of droplets containing cells to the total droplets is therefore 6 % in this experiment.

### 2.2 Study of alginate gel modification of anti-BTN2A2

**Fig. 2:**
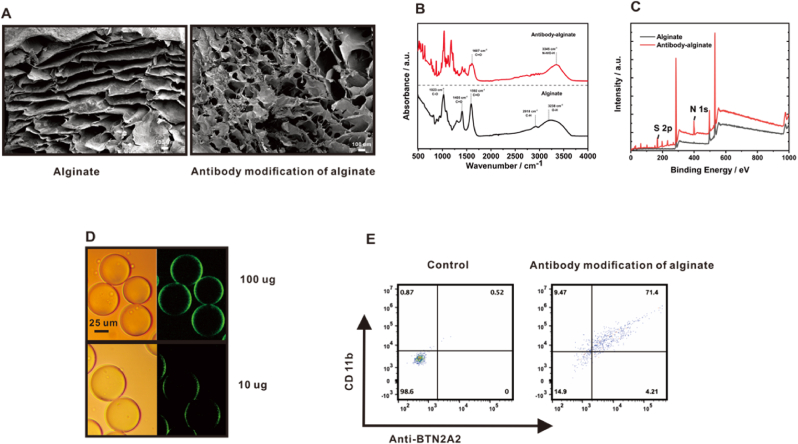
(A) SEM image of alginate modified anti-BNT2A2 nanoantibody. (B) FTIR spectra of alginic acid and anti-BNT2A2 modified alginic acid. (C) XPS analysis of alginate and anti-BNT2A2 modified alginate. (D) Bright field and fluorescence images of gel droplets modified with two masses of FITC-anti-BNT2A2. (E) Functional verifications of antibodies on alginate gels using flow cytometry.

Alginate hydrogels represent a versatile and highly adaptable biomaterial with substantial potential for biomedical applications. Alginate is a whole family of linear copolymers containing blocks of (1,4)-linked *β*-D-mannuronate (M) and α-L-guluronate (G) residues. Alginate all contain chemical modification sites that allow precise adjustment of functional modifications to the requirements of specific cell types [32]. However, conventional large molecular weight antibodies may lose their original biological activity when chemically modified, and there are strict limitations on conditions such as reaction time, temperature, and pH, resulting in low modification efficiency. Therefore, Nanobody Technology was utilized in this experiment. Nanobody Technology (NAT) is a biotechnology based on single-domain antibodies or called nanobodies. Nanobodies are typically only 12–15 kDa, much smaller than conventional antibodies, which makes it easier for them to penetrate tissues and get inside cells. At the same time, nanobodies exhibit higher stability under extreme conditions (e.g., high temperature, low pH) [33]. As shown in Fig. 2A, the SEM of alginate gel and anti-BNT2A2 nanoantibody modified alginate gel. This was followed by FTIR characterization, and the FTIR features of the alginate gel samples before and after anti-BNT2A2 nanoantibody modification are shown in Fig. 2B. With antibody doping, the O-H absorption peak near 3384 cm^−1^ is red-shifted to 3345 cm^−1^, which is due to the introduction of a large number of antibody N-H groups to enhance the intermolecular hydrogen bonding of the sample. The increase in the relative intensity of the absorption peak at 1607 cm^−1^ correlates with the C=O stretching vibration mode of the amide group, indicating that the activated alginate reacted with the amine group to form an amide structure and the antibody was successfully modified onto the surface of the alginate gel. XPS spectroscopy revealed the elemental composition in alginate gels and antibody-modified alginate gels (Fig. 2C). The results showed that N 1s peaks and S 2p peaks appeared in the antibody-modified alginate gel because antibodies usually contain nitrogen elements, and also, antibodies contain sulfur-containing amino acids. The fluorescence emitted by the modified antibody on the droplet can also be clearly seen through an inverted fluorescence microscope (Fig. 2D). Utilizing the stability of nanobodies at extreme temperatures, we successfully obtained gel droplets with varying antibody qualities by modulating the chemical coupling time and temperature (Fig.S3B, C and D). Meanwhile, flow cytometry characterization experiments were performed using the antibody-modified alginate gel (Fig. 2E).

Mechanical properties of gels were studied after modification of antibodies. As shown in Fig. S4, antibody modification, cell encapsulation and increase in CMP kept G’ > G” constant, which indicates that the system has normal hydrogel properties before and after. Antibody modification and CMP increase also made G′ higher, indicating that antibody modification and CMP increase can improve the rigidity and deformation resistance of the gel (Figs. S4B and C). When both the antibody modification and the increase in CMP also made G″ higher, it indicates that the antibody modification and the increase in CMP improved the gel viscosity (Figs. S4B and C). Alginate hydrogels encapsulated individual cells. The results showed that the G′ and G″ values of the microgels were lower than those of the unencapsulated alginate hydrogels, which may be due to the presence of cells affecting the mechanical properties of the gels (Fig. S4A). Combining all the influencing factors, ABGD has improved rigidity, deformation resistance and adhesion over alginate hydrogel (Fig. S4D). The experiments verified that the alginate gel modified using antibodies could be used as a fluorescent antibody for T-cell flow cytometry, indicating that the antibodies on the surface of the alginate gel functioned effectively.

### 2.3 Impact of Anti-bnt2a2-modified Alginate gel droplets on cellular Activity

**Fig. 3:**
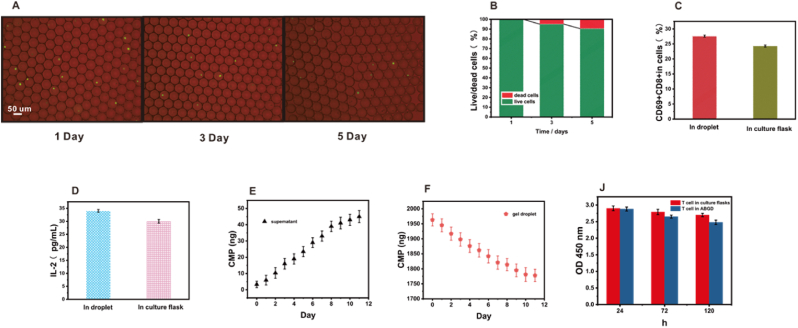
(A) Images of cell survival in droplets on days 1, 3, and 5. Cells were stained with Calcein AM. (B) Cell survival in droplets on days 1, 3, and 5. (C) Droplets and culture flasks were incubated for one day each, and the percentage of CD69^+^ CD8^+^ T cells in the cells. (D) Droplets and culture flasks were incubated for one day each, and the concentrations of IL-2 secreted in the respective cells. (E) Levels of CMP in supernatants at different times. (F) Levels of CMP in droplets at different times. (J) CCK-8 assay of T cells isolated from blood in culture flasks and in ABGD. IL-2 in gel droplets and supernatants using ELISA. CMP within gel droplets and in supernatants were determined by BSA. The number of cells was 10^6^.

According to the above method, T cells isolated from blood were encapsulated in anti-BNT2A2 modified alginate gel droplets using the microfluidic chip. An ABGD cell preparation was prepared. The function of ABDG cell preparation was explored in vitro. The initial focus was on investigating the impact of antibody-modified gel droplets on cellular activity. The gelation of droplets required calcium ions for cross-linking, and calcium ion concentration had an effect on cell activity. Therefore, the concentration of calcium ions added was carefully controlled. As shown in Fig. S5A, with the gelation time controlled at 10 min, the survival rate of the cells is drastically reduced with the increase of the concentration of added calcium ions. Therefore, a calcium ion concentration of 0.1 mM was chosen to gel the alginate in this experiment. As for the quality of the droplet-modified antibody, it had little effect on the activity of the cells, which may be due to the small molecular weight of the nanoantibody, which will not affect the material exchange of the droplet, and the BTN2A2 antibody itself had no effect on the cells (Fig. S5B). As shown in Fig. 3A and B, droplets were gelatinized at a concentration of 0.1 mM calcium ions using selected surface-modified antibodies of 100 μg alginate. The survival rate of its encapsulated cells at five days was 86 %. CD69 positivity serves as a crucial indicator of immune cell activation and functional initiation [34]. T cells within droplets and those in conventional culture flasks were tested. As depicted in Fig. 3C, the proportion of CD69^+^ CD8^+^ T cells exhibited minimal difference between the two conditions. The secretion of IL-2 by T cells was also assessed. The levels of IL-2 produced by T cells within the droplets were comparable to those in conventional culture flasks, indicating that the encapsulation in droplets did not significantly impact the proliferative and differentiation capabilities of the T cells (Fig. 3D). To enhance the infiltration of T cells into the tumor microenvironment, CMP was encapsulated within gel droplets. CMP, which mimics collagen peptides, is capable of replicating the triple-helix structure of natural collagen—a critical signal for cell recognition and adhesion. CMP can bind to integrins on the cell surface and enhance adhesion between cells and the stroma, and this enhanced adhesion helps cells better colonize and migrate in the tumor microenvironment. In addition, CMP enhance cell motility by activating intracellular signaling pathways. By providing a similar structural framework, CMP can promote cell adhesion and migration [35]. Previous studies had demonstrated that the release of proteins from alginate gels, when their molecular weights were similar, depends on their effective net charge, with charge-charge interactions predominantly governing the release rates of these molecules [15]. Alginate gels possessed a complex anionic network structure. Given that CMP (4 kD) had a pI of 8.9 and exhibits a positive charge in a solution at pH 7.2, its release rate was exceptionally slow due to electrostatic interactions (Fig. 3E and F). This slow release within the droplets more accurately mimicked the conditions of real cells, thereby facilitating the infiltration of T cells into the tumor microenvironment. The CCK-8 test of isolated T cells in culture flasks and ABGD as shown in Fig. 3J, where there was little change in T cell activity over 1, 3, and 5 days. The activity of the cells remained good for 5 days, and the modification of the antibody had little effect on the activity of the cells.

### 2.4 Research on the treatment of melanoma based on ABGD cellular preparations

**Fig. 4:**
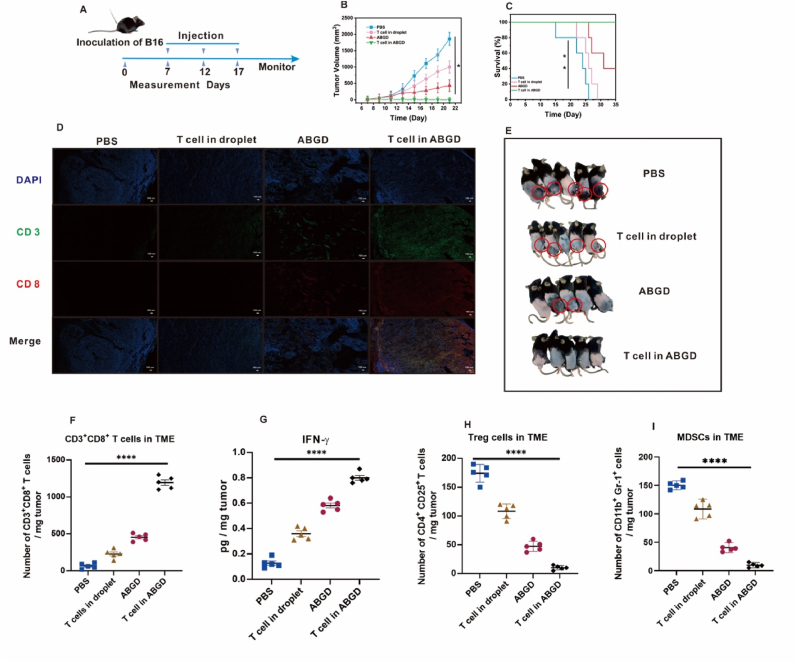
(A) Schematic illustration of the schedule for tumor model implantation and ABGD cell preparations-mediated therapy. (B) Growth curves of primary tumors in B16-tumor-bearing mice after different treatments. (C) Survival data of different groups of mice challenged with B16 tumors. (D) At 12-day, immunofluorescence staining of CD3^+^ CD8^+^ T cells for tumor tissues sections of B16 tumor-bearing mice (scale bar: 100 μm). (E) Pictures of tumors in mice on day 17. Mouse tumor sites are marked with red circles. (F) Numbers of tumor infiltrating CD3^+^ CD8^+^ T cells were measured on day 12. (G) Concentrations of IFN-γ were measured in tumors using ELISA. (H) Numbers of tumor infiltrating Treg cells were measured on day 12. (I) Numbers of tumor infiltrating MDSCs were measured on day 12. ∗p < 0.05; ∗∗p < 0.01; ∗∗∗∗p < 0.0001. (For interpretation of the references to colour in this figure legend, the reader is referred to the Web version of this article.)

ABGD cell preparations based on microfluidic chip preparation were first used to verify whether they could treat melanoma. A tumor model was established by subcutaneously injecting 3 × 10^6^ B16F10 cells into C57BL/6 mice, designated as the group α and group β. Seven days post-injection, melanoma growth was evident in the mice of group α. Subsequently, the mice in group α were euthanized, and their blood was collected. T cells were then isolated from the collected blood using an integrated chip for the preparation of ABGD cell formulations. Subsequently, melanoma-bearing mice in the group β received injections of PBS, droplet-encapsulated T cells (T cells in droplet), ABGD (No cells in the droplet), and ABGD-encapsulated T cells (T cells in ABGD) as control treatments. These injections were administered every 5 days, totaling three injections, with subsequent observation of the mice (Fig. 4A). As shown in Fig. 4B, tumor growth was inhibited using the ABDG cell preparation (i.e., T cells in ABGD), and the tumor volume was maintained at 6.4 mm^3^ for 12 days. By day 17, the tumors in some of the mice had completely regressed (Fig. 4E). The survival rate of the mice in the ABDG cell preparation-treated group was 100 % within 35 days (Fig. 4C). Immunohistochemistry of tumor sections was done on day 12 of treatment and it was seen that T-cell infiltration was submitted to be high in mice that were treated utilizing the ABGD cell preparation (Fig. 4D). There were about 900 CD3^+^CD8^+^ T cells per milligram of tumor and secreted IFN-γ was also higher (Fig. 4F and G), while Treg cells and MDSCs were suppressed (Fig. 4H and I). The data showed that treatment using only ABGD could also inhibit tumor growth, but a single immunosuppressant could not work consistently, and the mice tumors recurred once the injection treatment was stopped (Fig. 4C). It might be due to the inability to activate the immune response in vivo. From Fig. S6 A and B, it could be found that mice treated using ABGD had less IFN-γ secretion and fewer T cells in the spleen, indicating that the immune response in vivo was not activated. Therefore, ABGD cell preparations prepared using integrated microarrays can increase tumor infiltration by T cells and inhibit Treg cells and MDSCs in tumors, acting as a kind of combination therapy of adoptive cell immunotherapy and immunosuppressants. As shown in Fig. S9, TNF-α secretion was inhibited after treatment, and the inhibition correlated with a slowing of tumor growth.

### 2.5 Study of ABGD cell preparations for the treatment of pancreatic cancer

In this study, the treatment experiments in melanoma-bearing mice demonstrated notable efficacy. This result is likely due to the ability of CD8^+^ T cells from the peripheral blood of melanoma patients to specifically recognize melanoma cells, possessing T cell antigen receptors (TCRs) similar to those of tumor infiltrating CD8^+^ T cells. Our designed methodology holds promise in reducing the barriers encountered by T cells during tumor cytotoxicity, thereby effectively overcoming the immunosuppressive effects imposed by the tumor microenvironment. Building on these findings, we further explored whether peripheral blood T cells from other patients, which possess tumor infiltrating CD8^+^ T cell antigen receptors, could be harnessed using our strategy for the treatment of different types of tumors. To this end, we selected pancreatic cancer as a model for validation. Pancreatic cancer, often termed the 'king of cancers,’ is characterized by its extremely poor treatment outcomes, with a five-year survival rate ranging from only 7 %–11 % [36]. The low rate of surgical respectability, coupled with its insensitivity to radiotherapy and chemotherapy, severely limits therapeutic options. Moreover, the highly immunosuppressive microenvironment of pancreatic cancer poses significant challenges for immunotherapy, with various endogenous and exogenous mechanisms contributing to suboptimal treatment efficacy. Previous studies have demonstrated the presence of antigen receptors similar to those of tumor-infiltrating T cells in the peripheral blood of pancreatic cancer patients [37].

**Fig. 5:**
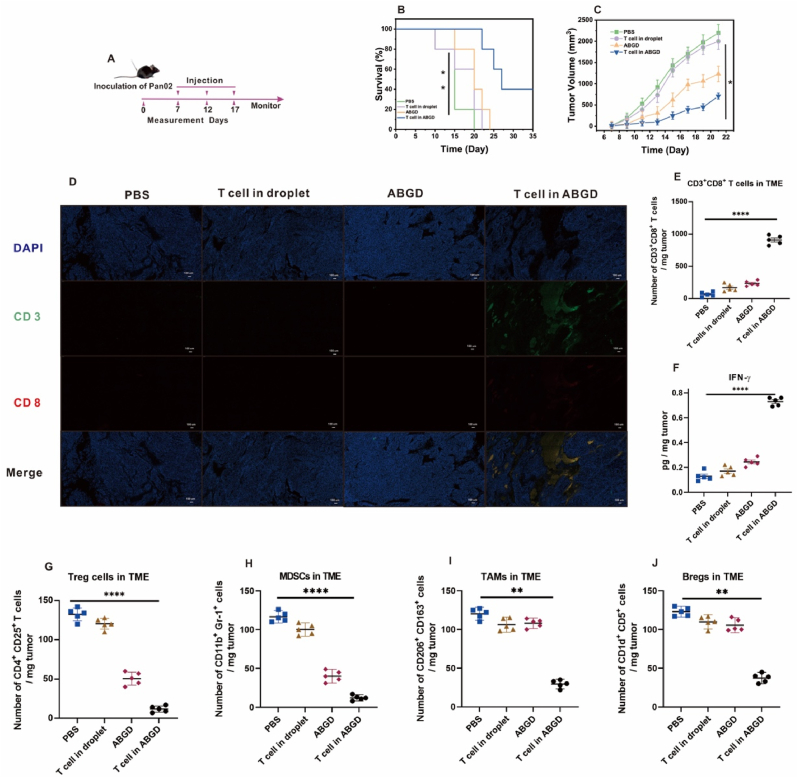
(A) Schematic illustration of the schedule for pancreatic cancer model implantation and ABGD cell preparations-mediated therapy. (B) Survival data of different groups of mice challenged with Pan02 tumors. (C) Growth curves of primary tumors in Pan02-tumor-bearing mice after different treatments. (D) At 12-day, immunofluorescence staining of CD3^+^ CD8^+^ T cells for tumor tissues sections of Pan02-tumor-bearing mice (scale bar: 100 μm). (E) Numbers of tumor infiltrating CD3^+^ CD8^+^ T cells were measured on day 12. (F) Concentrations of IFN-γ were measured in tumors using ELISA on day 12. (G) Numbers of tumor infiltrating Treg cells were measured on day 12. (H) Numbers of tumor infiltrating MDSCs were measured on day 12. (I) Numbers of tumor infiltrating TAMs were measured on day 12. (J) Numbers of tumor infiltrating Bregs were measured on day 12. ∗p < 0.05; ∗∗p < 0.01; ∗∗∗∗p < 0.0001.

Using a strategy analogous to that employed in the treatment of melanoma-bearing mice, C57BL/6 mice were subcutaneously injected with 3 × 10^6^ Pan02 cells to establish a tumor model, which were then divided into α and β groups. T cells were subsequently isolated from collected blood using an integrated chip for the preparation of ABGD cellular formulations. The melanoma-bearing mice in the β group were administered injections of PBS, droplet-encapsulated T cells (T cells in droplets), ABGD (droplets without cells), and ABGD-encapsulated T cells (T cells in ABGD) as control treatments. Injections were administered every 5 days, totaling three injections, followed by observation of the mice (Fig. 5A). As shown in Fig. 5B, mice treated with ABGD cellular preparations exhibited a 40 % survival rate over a period of 35 days. Within the first 22 days of treatment, tumor volume was effectively controlled (Fig. 5C). Immunofluorescent analysis of tumor sections at day 12 post-treatment revealed that neither T cells alone nor ABGD alone could promote T cell infiltration (Fig. 5D). In contrast to melanoma treatment, the use of ABGD alone was observed to facilitate T cell infiltration (Fig. 4D). Within the context of pancreatic cancer, treatment with ABGD alone resulted in CD3^+^ CD8^+^T cell infiltration rate of 220 cells/mg (Fig. 5F), whereas in melanoma, the infiltration was more than double, approximately 500 cells/mg (Fig. 4F). It might be due to the fact that melanoma mainly regulates the direct action of Tregs and MDSCs to inhibit T-cell infiltration [38], whereas pancreatic cancer relies on multiple immunosuppressive cells and synergistic actions, including Tregs, MDSCs, tumor-associated macrophages (TAMs), and regulatory B cells (Bregs), among others [39]. The above conclusions were verified in this experiment, where ABGD alone was utilized for treatment of pancreatic cancer due to the droplet surface-modified BTN2A2 antibody, which inhibited only Tregs and MDSCs (Fig. 5G and H), but not TAMs and Bregs (Fig. 5I and J). As a result, an effective immune response could not be developed, as also shown by the detection of CD8^+^ T cells in the spleens of mice with pancreatic cancer, where the amount of CD8^+^ T cells in the spleen of mice treated using only ABGD was very small (Fig. S7). Experimental data indicate that the treatment of pancreatic cancer necessitates the synergistic action of BTN2A2 antibodies and T cells. The ABGD cell preparation effectively leverages this synergy between antibodies and T cells, demonstrating a certain degree of efficacy in the treatment of pancreatic cancer.

## 3 Conclusion

In this study, an anti-BTN2A2 modified gel droplets (ABGD) cell preparation was prepared using an integrated microfluidic chip. T cells were extracted directly from host blood and encapsulated in a gel droplet of surface modified BTN2A2 antibody containing collagen mimetic peptide (CMP). Subsequent to the return treatment, BTN2A2 antibody on the surface of the droplet inhibited the proliferation of Tregs and MDSCs in the tumor, and CMP within the droplet increased the ability of T cells to infiltrate the tumor microenvironment. This method had good curative results in mouse models of melanoma and pancreatic cancer. In conclusion, ABGD cell preparations prepared using microfluidic integrated microarrays may enable a new combined therapeutic strategy of non-invasive TIL and immune checkpoint inhibitors.

## 4 Experimental section

### 4.1 Materials

Negative photoresist SU-8 and the developer were purchased from MicroChem Co., USA. PDMS and curing agent were obtained from GE Toshiba Silicones Co., Ltd., Japan. MWCO 200 kDa dialysis membrane were purchased from Shanghai Qiaoxing Trading Co., China. Alginate were received Xi'an qiyue Biological Technology Co., Ltd., China. Fluorocarbon oil (HFE7500, Novec 7500 Engineered Fluid), 1H,1H,2H,2H-perfluoro-1octanol (PFO), bovine serum albumin (BSA), CalceinAM were purchased from Sigma-Aldrich. IFN-γ and TNF-α ELISA kits were purchased from eBioscience. The peptide GGYGGGPC(GPP)5GFOGER(GPP)5GPC, where O is hydroxyproline, was synthesized by Nanjing Peptide Valley Biotechnology Co., China. Anti-BTN2A2 nanobodies purchased from Kejing Biological Technology Co., Ltd. Fluorochromelabeled antibodies (CD11b, CD8, CD3, CD4, CD14, CD25, Gr-1, CD45, CD206, granzyme B, CD163, CD1 and CD5) were received from Sigma-Aldrich (St. Louis, MO, USA). Ultrapure water (18.2 MΩ cm) was obtained from a Millipore Milli-Q system. Sulfo-NHS, EDC, phosphate-buffered saline (PBS) and RPMI 1640 medium were purchased from Thermo Fisher.

### 4.2 Design and fabrication of microfluidics chip

Microfluidic devices were made with polydimethylsiloxane (PDMS, GE Toshiba Silicones Co., Ltd., Japan) employing conventional soft lithography techniques. A 100-μm-thick master mold was prepared on the silicon wafer using SU-8 2050 (Microchem, Westborough, MA, USA). To mold the PDMS device, a prepolymer and curing agent was mixed in a 10:1 ratio (w/w), vacuum degassed, and poured over the fabricated SU8 mold before curing at 75 °C for 4 h. The solidified PDMS part was then mildly uncovered from the mold, and a biopsy puncher (Harrwas UniCore™) was used to create the inlet and outlet holes. The PDMS replica was bonded to a glass slide (70 mm × 30 mm) after oxygen plasma treatment. The chip was extra baked at 100 °C for >20 h and utilized ultraviolet irradiation for 2 h for sterilization.

### 4.3 Production of antibody-modified alginate

Alginate was chemically modified using aqueous carbodiimide solution and the reaction scheme is shown in Fig. S2. The alginate solution was reconstituted in a MES solution (0.1 M MES, 0.3 M NaCl, pH 6.5). 0.38 g of EDC and 0.12 g of sulfo-NHS were added to the solution and stirred continuously at 30 °C for 20 h. Subsequently, 200 μg FITC-anti-BTN2A2 was added to the solution. The solution was stirred for 20 min, dialyzed for 3 days (MWCO 20 kDa dialysis membrane) and lyophilized, taking care to avoid light the entire time. Finally, flow cytometry was utilized to verify the function of alginate surface antibodies.

### 4.4 Isolation of lymphocytes in microfluidic chip

The chip (Fig. S1) was pumped through with the 1 % BSA solution to decrease cell and channel composition adhesion. Blood inlet and buffer inlet were connected to the syringe pump (PHD ULTRA™, Harvard Apparatus) respectively for driving the sample at a constant flow rate. Isolated cells were collected at the output and then stained with fluorescent antibody (anti-CD14-PE, anti-CD3-PC7, anti-CD11b-PC5.5 and anti-CD45-FITC). By measuring the lymphocyte numbers of the outputs, the separation efﬁciency was calculated with the following equation:

$$separation\;efficiency=\frac{{\mathrm{LN}}_{2}}{{\mathrm{LN}}_{1}+{\mathrm{LN}}_{2}+{\mathrm{LN}}_{3}}\text{,}$$

where LN_1_ was the number of lymphocytes collected from output I, LN_2_ was the number of lymphocytes collected from output II and LN_3_ was the number of lymphocytes collected from output III. In this experiment, the separation efficiency was the percentage of lymphocytes isolated from the blood to the total number of lymphocytes in the blood.

Separation purity was calculated according to the following equation:

$$composition\;of\;lymphocyte=\frac{\mathrm{LN}}{TN}\text{,}$$

where LN was the number of lymphocytes collected from output II, TN was the total number of leukocytes from output II. Separation purity was the percentage of lymphocytes to the total number of cells in the output II.

### 4.5 Preparation of ABGD cell preparations in microfluidic chip

All the blood samples were processed by chip within 24 h by injecting 1000 μL mice blood into the chip at a flow rate of 50 μL/min. The mice blood, buffer (2 wt% antibody-modified alginate, and 2 μg CMP) and fluorocarbon oil (HFE7500, Novec 7500 Engineered Fluid, 1 wt% surfactant [40]) were loaded into the different inlets, respectively, and perfused at a flow rate ratio of 1:10:10 using syringe pumps (PHD ULTRA™, Harvard Apparatus). **The gel-droplets encapsulated T cells was collected at output II. 0.05 vol% calcium ions was added to cause gelation of the droplets. Following gelation, the gel-droplets were carried into the aqueous medium by adding a 20 % (v/v) solution of** *1H,1H,2H,2H-perfluoro-1-octanol (PFO, Alfa Aesar)* **in the perfluorinated carbon oil** [41]**. After centrifugation, the oil phase was discarded, and the gel-droplet were collected and re-suspended** *into the medium* (Roswell Park Memorial Institute (RPMI) 1640) *for incubation in the incubator (*at 37 °C under 5 % CO_2_). The cells in the gel droplets were stained with 1 μM calcein-AM (Invitrogen, Carlsbad, CA, green fluorescence, live stain) at different time periods to observe the activity of cells. Fluorescence images were obtained with an inverted fluorescence microscope (TE2000-U, Nikon, Japan). Droplets of encapsulated CMP were dispersed into 400 μL of cell culture medium, and CMP in the droplets and supernatant were assessed daily. CMP within the gel droplets and in the supernatant were determined by the BSA method.

### 4.6 Cells and animals

B16F10 cells and Pan02 were purchased from China Type Culture Collection. The media for cell culture were bought from PAN biotech and Gibco. C57BL/6 mice (6–8 weeks, female) were purchased from Changsha Tianqin Biotechnology Co. and bred at the SPF Animal Lab of Guizhou Medical University. The study protocol was reviewed and approved by Guizhou Medical University Animal Care Welfare Committee. Melanoma and pancreatic peripheral blood T cells were isolated using our previously designed microfluidic chip.

### 4.7 In vivo anticancer efficacy evaluation

C57BL/6 mice (6–8 weeks) were subcutaneously injected with 3 × 10^6^ B16F10 cells. On day 7, mice were randomly divided into four groups (n = 7). C57BL/6 mice were subcutaneous injection with PBS (200 μL per mouse), T cells in deoplet (200 μL per mouse, 1 × 10^7^ cells per mouse), ABGD (200 μL per mouse) and T cells in ABGD (200 μL per mouse, 1 × 10^7^ cells per mouse), respectively. The tumor volume and body weight were strictly observed. Tumor volumes (V, mm^3^) were calculated using the following formula: (W^2^ × L)/2, where W (width) was the short perpendicular dimension, and L (length) was the longest dimension. Survival of mice was also monitored. At 12 days, tumor tissues of B16 tumor-bearing mice were removed. Immunofluorescence were done. CD3^+^ CD8^+^ T cells and MDSCs were measured by flow cytometry. Mice were sacrificed on day 22, the spleen of mice was removed, in which IFN-γ measured by ELISA and CD3^+^ CD8^+^ T cells were measured by flow cytometry.

The pancreatic cancer treatment experiment was similar to the appeal experiment. C57BL/6 mice (6–8 weeks) were subcutaneously injected with 3 × 10^6^ Pan02 cells. On day 7, mice were randomly divided into four groups (n = 7). C57BL/6 mice were subcutaneous injection with PBS (200 μL per mouse), T cells in deoplet (200 μL per mouse, 1 × 10^7^ cells per mouse), ABGD (200 μL per mouse) and T cells in ABGD (200 μL per mouse, 1 × 10^7^ cells per mouse), respectively. The tumor volume and body weight were strictly observed. Tumor volumes (V, mm^3^) were calculated using the following formula: (W^2^ × L)/2, where W (width) was the short perpendicular dimension, and L (length) was the longest dimension. Survival of mice was also monitored. At 12 days, tumor tissues of B16 tumor-bearing mice were removed. Immunofluorescence were done. CD3^+^ CD8^+^ T cells, MDSCs, TAMs and Breg were measured by flow cytometry. Mice were sacrificed on day 22, the spleen of mice was removed, in which CD3^+^ CD8^+^ T cells were measured by flow cytometry.

### 4.8 Statistical analysis

Statistical analysis was performed with Prism 7.0e (GraphPad Soft-ware). Statistical comparisons were performed using one-way ANOVA followed by Tukey's HSD multiple comparison post hoc test or two-way ANOVA followed by Sidak's multiple comparisons test, as indicated in the figure legends. Survival data were analyzed by using a log-rank test. Results were expressed as mean ± SD. Statistical significances are indicated as ∗p < 0.05, ∗∗p < 0.01, ∗∗∗p < 0.001, and ∗∗∗∗p < 0.0001.

## CRediT authorship contribution statement

**Yishen Tian:** Writing – review & editing, Writing – original draft, Visualization, Validation, Software. **Jingxuan Li:** Writing – review & editing, Writing – original draft, Visualization. **Na Yang:** Visualization, Validation, Project administration. **Yang Zhao:** Investigation. **Jiancao Zuo:** Software. **Hang Xiong:** Formal analysis. **Yiwen Pan:** Investigation. **Li Xiao:** Formal analysis. **Min Su:** Resources, Project administration. **Feng Han:** Investigation, Funding acquisition. **Zhixu He:** Funding acquisition, Data curation. **Rong Hu:** Funding acquisition, Formal analysis, Conceptualization.

## Declaration of competing interest

The authors declare that they have no known competing financial interests or personal relationships that could have appeared to influence the work reported in this paper.

## Data Availability

Data will be made available on request.
