# Supplementary material for: Integrated microfluidics-based construction of anti-BTN2A2 gel droplet cell preparations for non-invasive tumor-infiltrating lymphocyte therapy
Source: Mater Today Bio. 2025 Feb 4;31:101545. doi: 10.1016/j.mtbio.2025.101545 (PMC11869011; doi:10.1016/j.mtbio.2025.101545)
Supplement: Multimedia component 1 [file mmc1.docx]

**Supplementary Information**

**Integrated microfluidics-based construction of anti-BTN2A2 gel droplet cell** **preparations for non-invasive** **tumor-infiltrating lymphocyte therapy**

*Yishen Tian^1,2,4*^, Jingxuan Li^1,2,3*^, Na Yang^1^, Yang Zhao^4^, Jiancao Zuo^4^, Hang Xiong^4^, Yiwen Pan^1,4^, Li Xiao^1,4^, Min Su^1,2,4^, Feng Han^3,^* *^a)^, Zhixu He^1,5, a)^ and Rong Hu^1,2,4, a)^*

**Affiliations**

*^1^* Center for Tissue Engineering and Stem Cell Research, Guizhou Medical University, Guiyang, 550025, China.

*^2^*Key Laboratory for Research on Autoimmune Diseases of Higher Education schools in Guizhou Province, Guiyang, 550025, China.

^3^ Department of Neurosurgery, The Affiliated Hospital of Guizhou Medical University, Guiyang, 550004, China.

^4^ Department of Histology and Embryology, School of Basic Medical Sciences, Guizhou Medical University, Guiyang, 550025, China.

^5^ Department of Pediatric Hematology, The Affiliated Hospital of Guizhou Medical University

*^a)^* **Authors to whom correspondence should be addressed:** hanfeng0115@gmc.edu.cn, hzx@gmc.edu.cn and hurong@gmc.edu.cn

*** **These authors contributed to the work equally and should be regarded as co-first authors.**


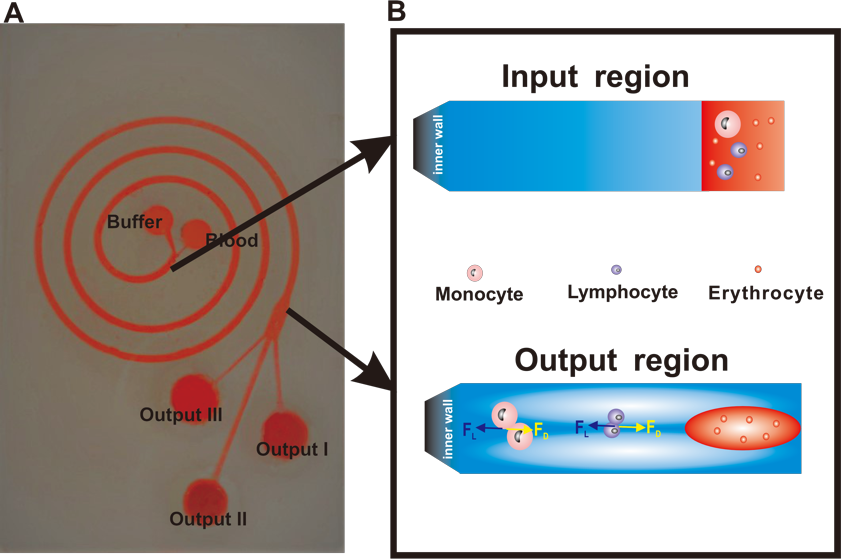


**Fig. S1.** (A) Top view of chip separation section (channel with red ink). (B) Schematic illustration of the separation principle.


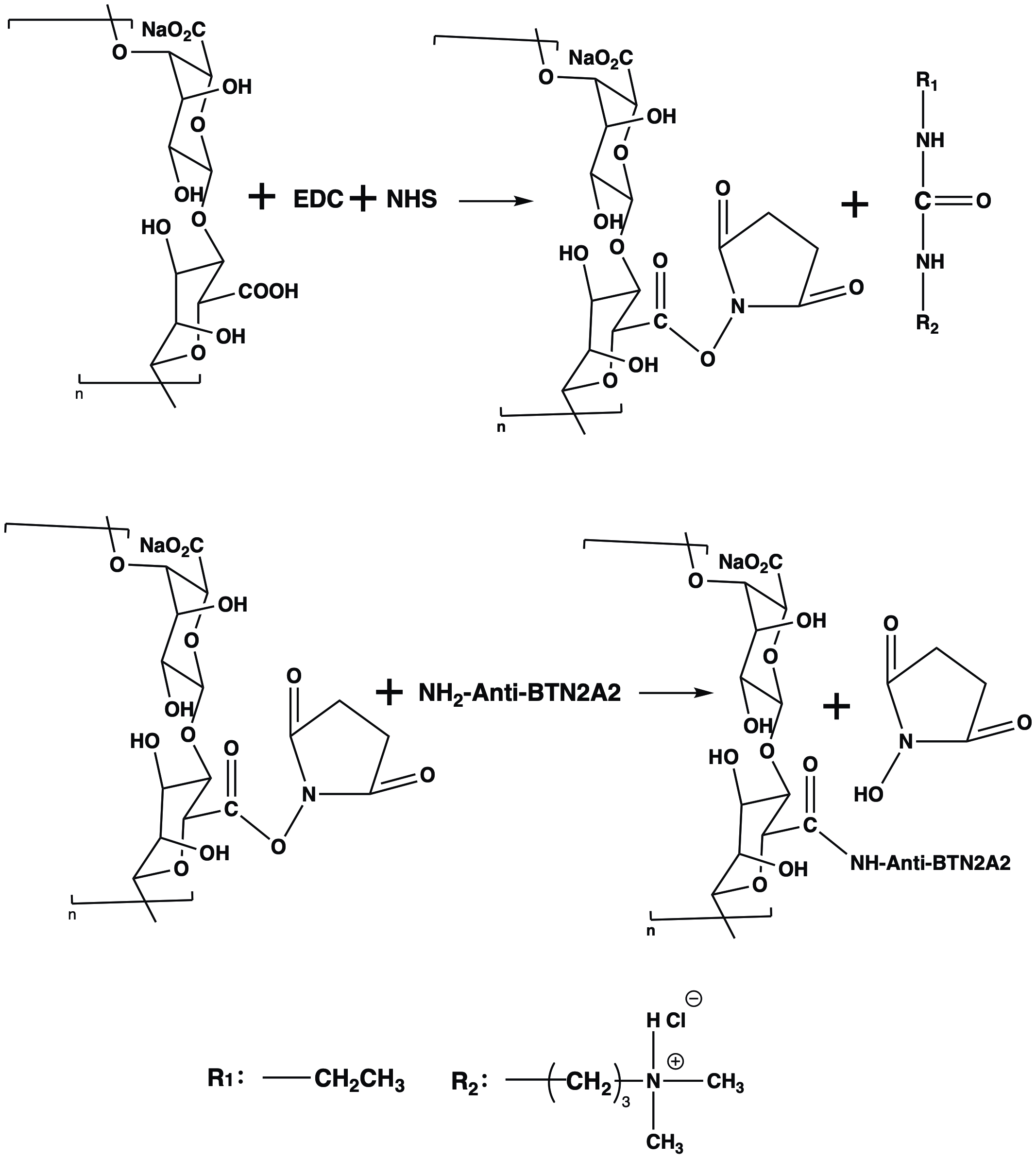


**Fig. S2.** Reaction scheme for antibody-modified alginate.


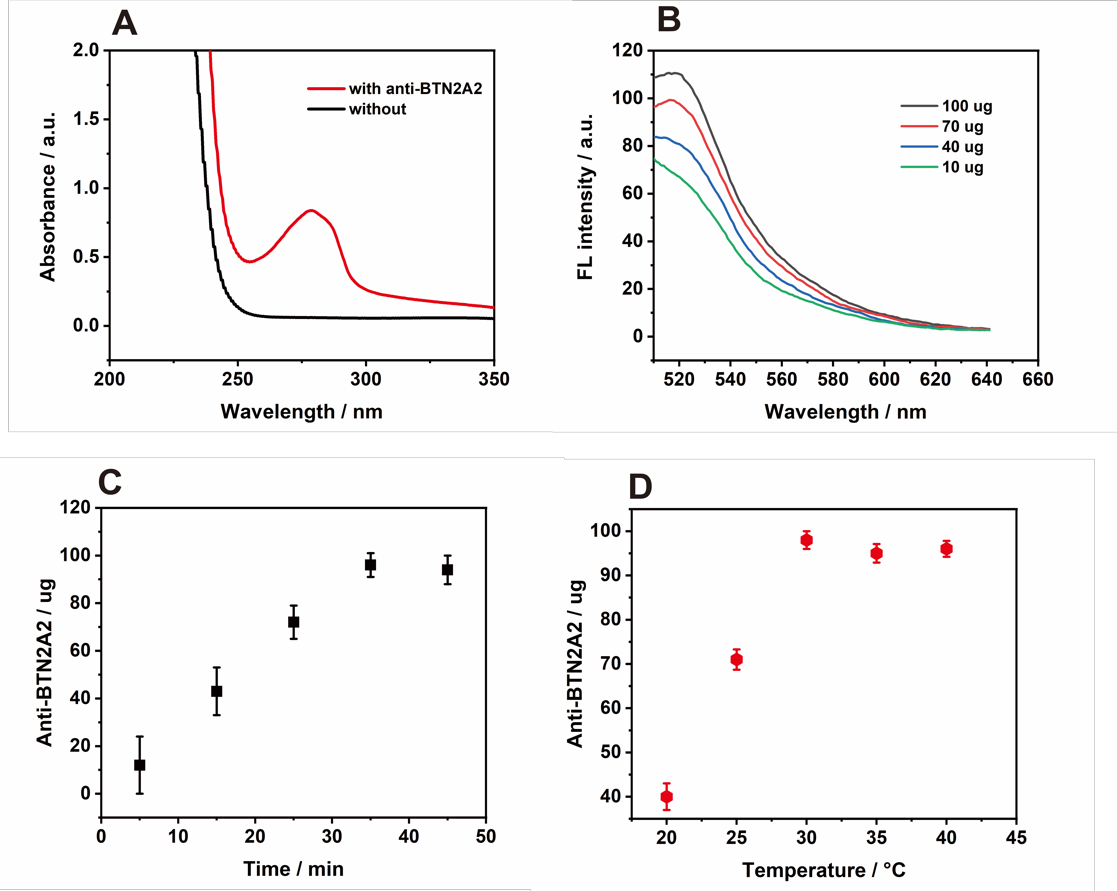


**Fig. S3.** (A) UV spectrum of antibody-modified alginate gel (red). (B) Fluorescence spectra of antibodies of different masses modified by alginate gel. (C)and (D) Time and temperature of chemical coupling, effect on modified antibody quality.


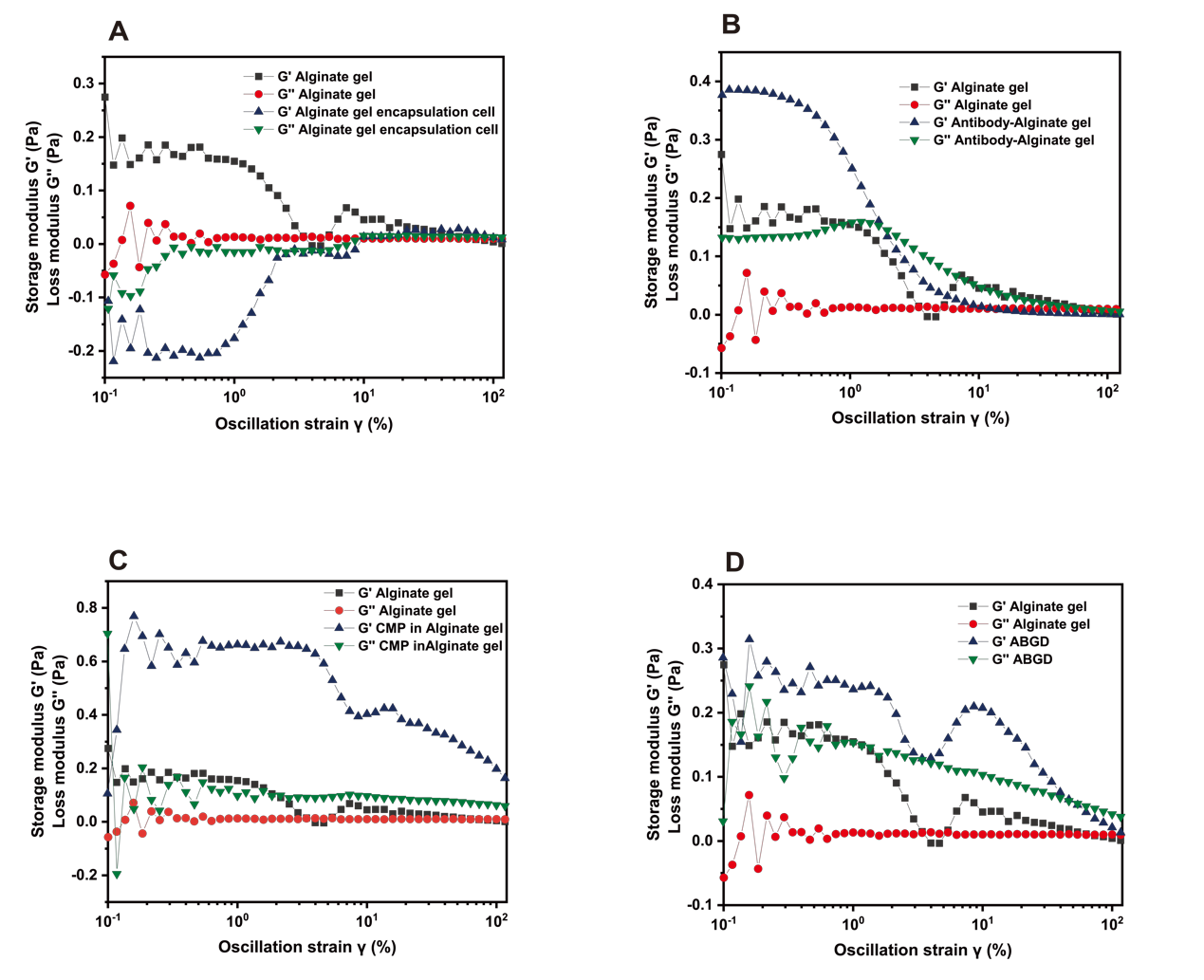


**Fig. S4.** Storage (G’) and loss (G’’) moduli versus Oscillation strain (γ). (A) Relationship between changes in G’, G’’ and γ of alginate gel and alginate gel after encapsulation of cells. (B) Relationship between changes in G’, G’’ and γ of alginate gels and antibody-modified alginate gels. (C) Relationship between changes in G’, G’’ and γ of alginate gels and alginate gels with increased CMP. (D) Relationship between changes in G’, G’’ and γ of alginate gels and ABGD. T=37$℃$, Frequency 1 Hz.





**Fig. S5.** (A) Effect of calcium ion concentration on cell viability during gelation. (B) Effect of the quality of alginate gel droplet-modified antibodies on cellular activity.





**Fig. S6.** (A) At 20 d after injection, ELISA analysis of secretion of IFN- γ in the spleen of mice. (B) The percentage of CD3 ^+^ CD8 ^+^ T cells in the spleen by flow cytometry. Values represent mean ± SDs (n = 5) from at least three independent experiments.


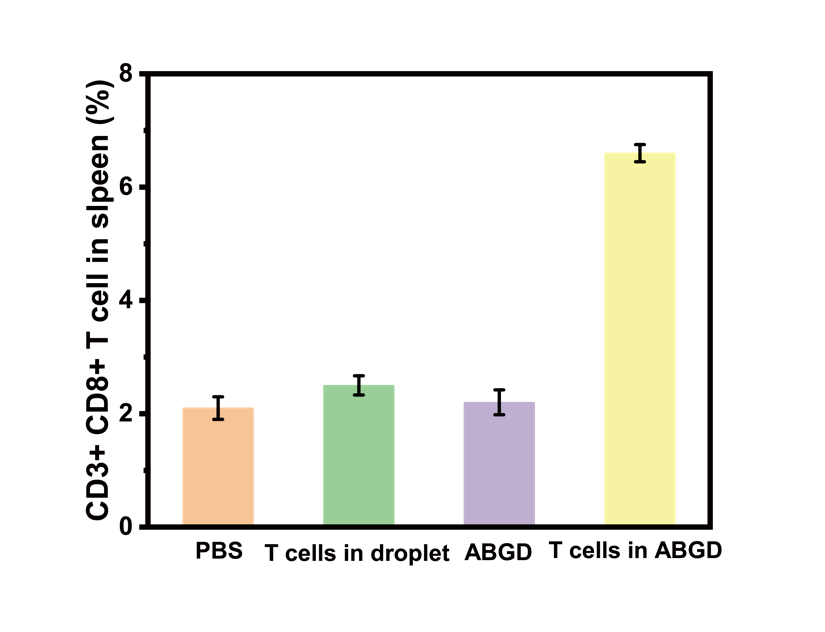
 A

**Fig. S7.** (A) The percentage of CD3^+^ CD8^+^ T cells in the spleen of mice with pancreatic cancer by flow cytometry. Values represent mean ± SDs (n = 5) from at least three independent experiments.


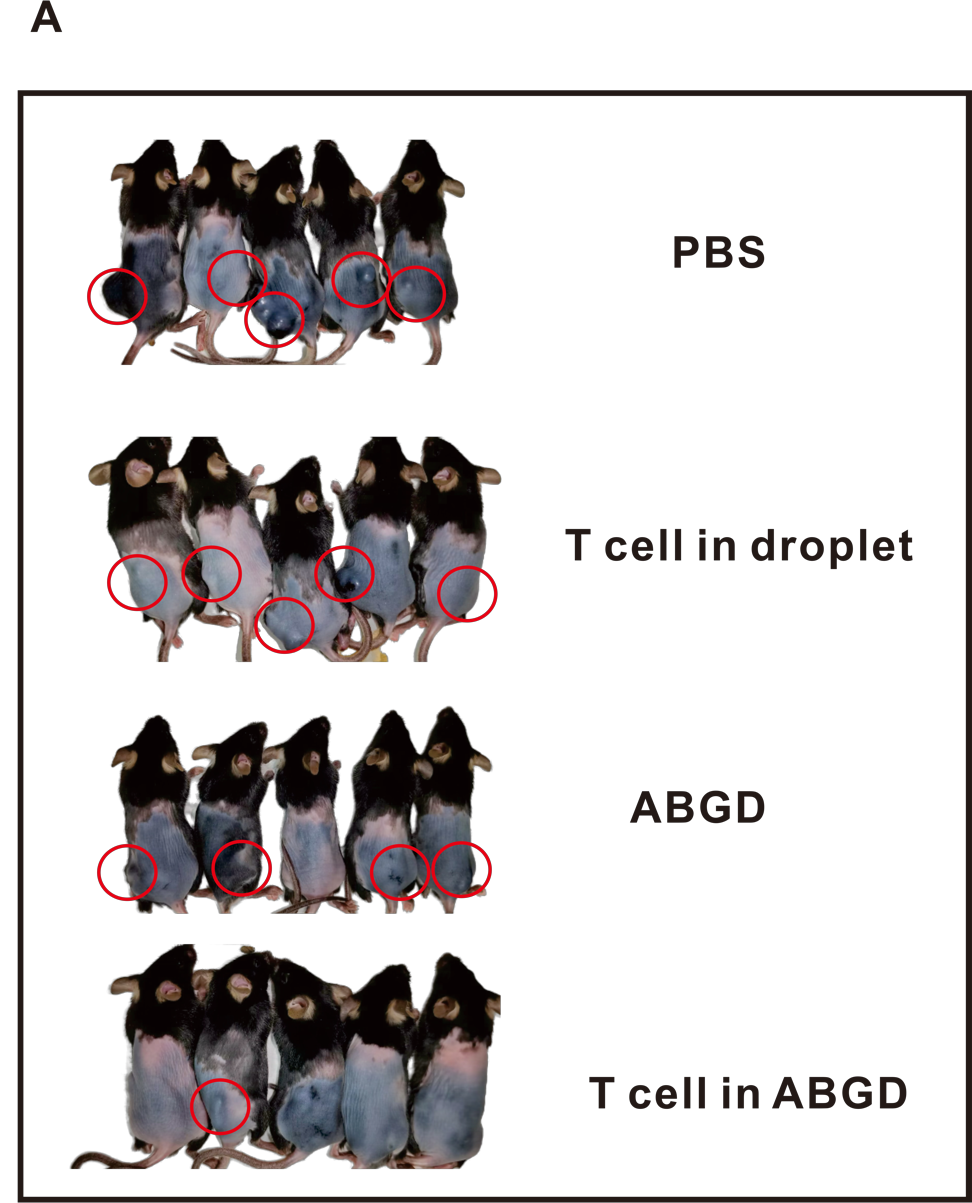


**Fig. S8.** (A) Pictures of tumors in mice with pancreatic cancer on day 17. Mouse tumor sites are marked with red circles.


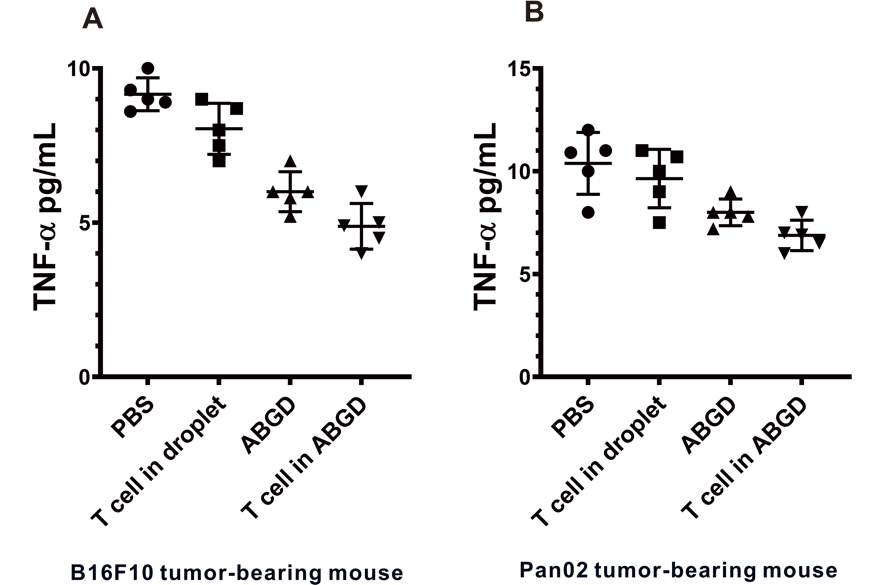


**Fig. S9.** (A) TNF-α levels in B16F10 tumor-bearing mouse after treatment. (B) In TNF-α levels in Pan02 tumor-bearing mouse after treatment.
